# Circular RNA MYLK promotes tumour growth and metastasis via modulating miR‐513a‐5p/VEGFC signalling in renal cell carcinoma

**DOI:** 10.1111/jcmm.15308

**Published:** 2020-04-27

**Authors:** Jianfa Li, ChenChen Huang, Yifan Zou, Jing Yu, Yaoting Gui

**Affiliations:** ^1^ Guangdong and Shenzhen Key Laboratory of Male Reproductive Medicine and Genetics Institute of Urology Peking University Shenzhen Hospital Shenzhen‐Peking University‐the Hong Kong University of Science and Technology Medical Center Shenzhen China; ^2^ Anhui Medical University Hefei China; ^3^ Department of Laboratory Medicine Peking University Shenzhen Hospital Shenzhen China; ^4^ Department of Urology The Affiliated Luohu Hospital of Shenzhen University Shenzhen China

**Keywords:** circMYLK, miR‐513a‐5p, renal cell carcinoma, therapeutic target, VEGFC

## Abstract

Growing evidence indicates that circular RNAs (circRNAs) are promising biomarkers, as they play significant roles in the development of various cancers. The circular RNA MYLK (circMYLK) has been reported to be involved in the development of malignant tumours, including liver, prostate and bladder cancers. Nevertheless, the biological function of circMYLK in renal cell carcinoma (RCC) remains unclear. In this study, we observed that circMYLK is notably up‐regulated in RCC. Increased circMYLK expression led to a larger tumour size, distant metastasis and poor prognosis of RCC patients. Moreover, circMYLK silencing repressed RCC growth and metastasis in vitro and in vivo. Mechanistically, circMYLK can capture miR‐513a‐5p to facilitate VEGFC expression and further promote the tumorigenesis of RCC cells. In summary, our findings demonstrate that circMYLK has an oncogenic role in RCC growth and metastasis by modulating miR‐513a‐5p/VEGFC signalling. Thus, circMYLK has potential as a diagnostic biomarker and therapeutic target in the treatment of RCC.

## 1 INTRODUCTION

Renal cell carcinoma (RCC) is one of the most malignant cancers and has a high mortality rate. It is estimated that more than 400 000 new RCC cases and 170 000 cancer‐related deaths occurred in 2018 worldwide.^1^ Partial or radical nephrectomy is suitable for early stage of RCC. However, approximately a third of RCC patients are diagnosed at the advanced stage at the primary diagnosis, a stage at which the overall survival of RCC patients is extremely low despite active treatment.^2^ Therefore, the identification of novel molecular mechanisms involved in RCC progression and effective therapeutic targets of RCC are urgently needed.

Circular RNAs (circRNAs) are a novel type of non‐coding RNA characterized by a covalently closed loop without a 5′ cap or 3′poly A tail. Compared to linear RNA, circRNAs are stable and not easily degraded by RNase R treatment.^3^ With the advent of high‐throughput sequencing, numerous circRNAs have been identified in various cell lines and tissues.^4^ Recently, the results of numerous studies have suggested that circRNAs participate in the development and progression of various cancers by regulating different biological processes, including cell differentiation, metastasis, proliferation, apoptosis, drug resistance and energy metabolism.^5^, ^6^, ^7^, ^8^ For instance, circTADA2A promotes the proliferation and metastasis of osteosarcoma cells by sponging miR‐513a‐5p to up‐regulate the expression of CREB3.^9^ In gastric cancer, circPSMC3 inhibits cell proliferation and metastasis in vitro and in vivo by acting as a competitive endogenous RNA (ceRNA) for miR‐296‐5p.^10^ In RCC, circAKT3 suppresses cell migration and invasion by modulating the miR‐296‐3p/E‐cadherin axis.^11^ Moreover, circRAPGEF5 functions as a sponge of miR‐27a‐3p to facilitate TXNIP expression and then suppresses the proliferation and migration of RCC cells in vitro and in vivo.^12^ A previous study suggested that circRNA MYLK (circMYLK) acts as a ceRNA to promote the proliferation and metastasis of bladder cancer by modulating the VEGFA/VEGFR2 signalling pathway.^13^ In prostate cancer, circMYLK can suppress miR‐29a expression to promote cell proliferation, migration and invasion.^14^ However, the pathological and biological functions of circMYLK in RCC remain unclear.

In this study, we observed that circMYLK was significantly increased in RCC tissues compared with that observed in matched adjacent normal tissues in a cohort of 71 RCC patients, and its expression was positively correlated with larger tumour size, distant metastasis and poor prognosis. Further experiments demonstrated that knockdown of circMYLK suppressed the proliferation and metastasis of RCC cells in vitro and in vivo. Mechanistically, we discovered that circMYLK was primarily distributed in the cytoplasm and it acts as a ‘miRNA sponge’ to positively modulate VEGFC expression in a ceRNA‐dependent manner. In addition, overexpression of VEGFC reversed the circMYLK silencing‐mediated suppression of RCC cell proliferation and migration. Taken together, our results revealed that circMYLK may serve as an oncogene in the progression of RCC and that it may be a promising diagnostic biomarker and therapeutic target in the treatment of RCC.

## 2 MATERIALS AND METHODS

### 2.1 Patient tissue specimens

Seventy‐one RCC tissues and matched adjacent normal renal tissues were collected from RCC patients who underwent surgery. We had received permission from the Ethical Committee of Perking University Shenzhen Hospital before tissue collection. All RCC patients agreed that their tissues could be used to this study and paper presentations.

### 2.2 Cell lines

HK‐2 cell and human RCC cell lines (ACHN, 786‐O and Caki‐2) were purchased from the American Type Culture Collection (ATCC). The HK2 cell was cultured in DMEM medium (Gibco) supplemented with 10% foetal bovine serum (Gibco) and 1% penicillin/streptomycin (Gibco). ACHN, 786‐O and Caki‐2 cells were grown in RPMI‐1640 medium (Gibco) supplemented with 10% FBS and 1% penicillin. All of these cell lines were grown in an incubator under an atmosphere with 5% CO_2_ at 37°C.

### 2.3 RNA extraction and quantitative real‐time PCR (qRT‐PCR) assay

Total RNA derived from RCC tissues and cell lines was extracted using TRIzol reagent (Invitrogen). qRT‐PCR was conducted utilizing a standard SYBR Green PCR Kit (Takara), and the reactions were performed using a Roche LightCycler^®^ 480II PCR instrument in triplicate. GAPDH or U6 small nuclear RNA was used as internal controls. The sequences of primers used in this study are shown in Table S1.

### 2.4 Oligonucleotide transfection

The shRNA‐circMYLK (sh‐circMYLK) and corresponding control oligonucleotide were synthesized by GenePharma. The sequence of sh‐circMYLK was as follows: TAGAAGACCATGGGGGATGTCAAGAGCATCCCCCATGGTCTTCTATTTTTT. sh‐circMYLK was synthesized and cloned into the vector pGPU6/GFP/Neo. miR‐513a‐5p mimics and the corresponding control oligonucleotide were obtained from RiboBio. The circMYLK sequence was synthesized by GenePharma and cloned into the vector pcDNA3.1, which that possesses the front and back circular frames. The coding sequence (CDS) of VEGFC was cloned into the vector pcDNA3.1. All transfections were performed using Lipofectamine 3000 reagent (Invitrogen) with a final concentration of 60 nmol/L miRNA mimics and 3 µg of plasmids according to the manufacturer's instructions. For stable transfection, ACHN cells were infected with lentivirus expressing sh‐circMYLK and then selected with 3 μg/mL puromycin for 2‐3 weeks.

### 2.5 Cell proliferation assay

Cell counting Kit‐8 (CCK‐8) (Beyotime Institute of Biotechnology) and clone formation assays were conducted to measure cell proliferation. For the CCK‐8 assay, the transfected RCC cells were grown in 96‐well plates, and the absorbance of the transfected RCC cells was determined using a microplate reader at 450 nmol/L after 0, 24, 48, 72 and 96 hours. For the clone formation assay, 1000 transfected RCC cells per well were grown in 6‐well plates for 2‐3 weeks. Subsequently, the cell colonies were stained with 0.1% crystal violet and then washed with 33% glacial acetic acid.

### 2.6 Wound‐healing assay

RCC cells were seeded in 6‐well plates after transfection. A wound field was created by a pipette tip when cells reached 90%‐100% confluence. Then, the damaged cells were grown in fresh medium without serum for 24 hours after being washed with PBS. Finally, the RCC cells were microscopically viewed and imaged at 5× magnification.

### 2.7 Transwell assay

Transwell assays were conducted to investigate the migratory and invasive capacities of RCC cells. In this assay, 2‐3 × 10^4^ cells were placed in an 8‐μm Transwell insert (Corning) coated with or without Matrigel in serum‐free medium (BD Biosciences) after transfection. After incubating for 24‐48 hours, the migrated and invasive cells were stained with 0.1% crystal violet and then microscopically visualized at 10× magnification. Finally, the Transwell inserts were soaked in 33% glacial acetic acid.

### 2.8 Western blotting assay

Transfected RCC cells were treated with PIPA lysis buffer (Beyotime) supplemented with protease inhibitor cocktail. Total protein from RCC cells was separated by 10% SDS‐PAGE and transferred to a PVDF membrane. Then, the membrane was blocked with 5% skim milk powder and then incubated with primary antibodies at 4°C for 12‐16 hours. Subsequently, the membranes were incubated with a secondary antibody at room temperature for at least 1 hour, and the resulting autoradiograms were analysed by densitometry with Quantity One software (Bio‐Rad).

### 2.9 RNA pull‐down assay

To pull‐down the miRNA captured by circMYLK, ACHN and 786‐O cells transfected with a circMYLK‐overexpressing plasmid were incubated with a biotin‐labelled circMYLK probe for 24‐48 hours. Then, TRIzol reagent was used to extract and purify the pull‐down products, and the bound miRNAs were quantified by qRT‐PCR.

### 2.10 Luciferase reporter assay

The luciferase reporter plasmids (the MT06 vector contained the circMYLK wild‐type (WT) or mutant (Mut) sequences, and the MT07 vector contained the VEGFA WT or Mut sequences) were synthesized by Genecopoeia. Subsequently, 293‐T cells were co‐transfected with the luciferase reporter plasmids and miR‐513a‐5p mimics. Relative luciferase activities were determined using a Dual Luciferase Reporter Kit (Promega) after transfection.

### 2.11 Tumour xenograft

For tumour xenograft assays, 10 4‐week‐old female BALB/c nude mice were randomly separated into two groups. The mice in each group were subcutaneously injected in the back with 5 × 10^7^ ACHN cells stably expressing sh‐circMYLK or negative control plasmids. The volume of all tumour xenografts was measured every week, and 6 weeks after injection, all mice were killed to assess tumour weight. Total RNA and protein from tumours were extracted to calculate gene expression. For the tumour metastasis xenograft, nude mice were tail vein injected with 2 × 10^7^ ACHN cells stably expressing sh‐circMYLK or negative control plasmids. Then, 2 months after injection, all mice were killed to measure the pulmonary metastatic foci.

### 2.12 Statistical analyses

All data from independent repeated trials are presented as the means ± standard deviation (SD) and analysed with SPSS 22.0 (SPSS). Student's *t* test was used to analyse group difference, and differences with *P* values < .05 were recognized as significant.

## 3 RESULTS

### 3.1 CircMYLK expression is augmented in RCC tissues and cell lines

**Figure 1:**
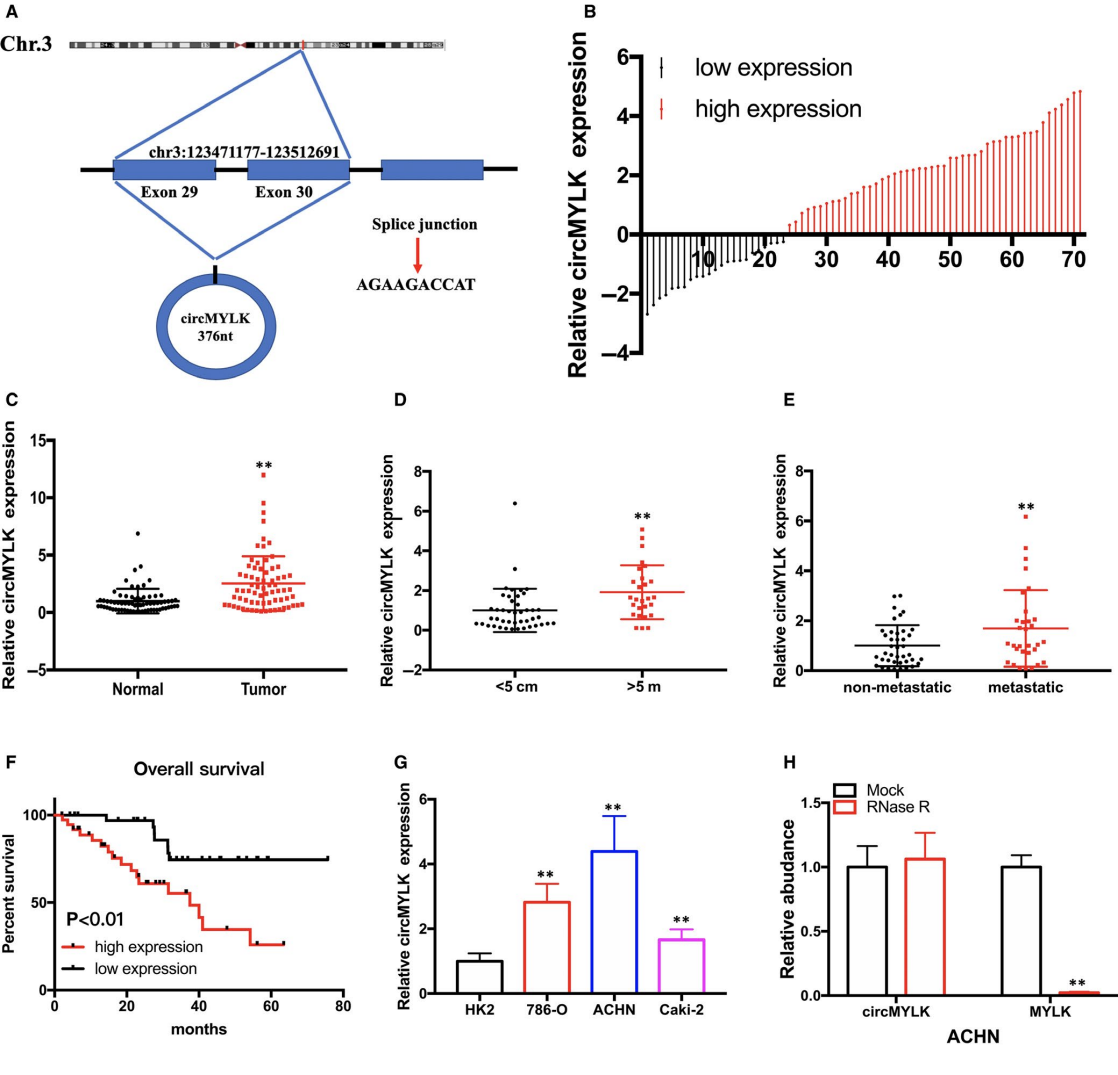
circMYLK expression is up‐regulated in RCC tissues and positively associated with poor prognosis. A, Schematic illustration showing the production of circMYLK. B and C, The level of circMYLK expression in RCC tissues and matched non‐tumour tissues is shown. D, The level of circMYLK expression in RCC with different tumour sizes. E, The level of circMYLK expression in RCC patients with or without distant metastasis. F, Kaplan‐Meier plots of the overall survival of RCC patients with high and low circMYLK expression. G, circMYLK expression was up‐regulated in RCC cells compared to that observed HK2 cells. H, qRT‐PCR was performed to measure the expression of circMYLK and MYLK mRNA in ACHN cells upon RNase R treatment. I, circMYLK was primarily localized in the cytoplasm. *P < .05 and **P < .01

Utilizing the Circbase Database, we determined that circMYLK is 376 nucleotides (nt) in length and is derived from back‐splicing of MYLK mRNA, and the gene is located on chr3:123471177‐123512691 (Figure 1A). qRT‐PCR was performed to detect circMYLK expression in 71 RCC tissue and matched non‐tumour tissue samples. As shown in Figure 1B and C, circMYLK was significantly up‐regulated in 66.2% (47 of 71) of RCC tissues. Red column represents relative high expression of circMYLK, and dark column represents relative low expression of circMYLK. Moreover, up‐regulated circMYLK was positively correlated with tumour size and distant metastasis of RCC tissues (Figure 1D‐E and Table 1). Using the data set of RCC patients, we observed that patients with high circMYLK expression levels had a poorer overall survival compared with those with low circMYLK expression levels (Figure 1F). Furthermore, our data showed that circMYLK expression was augmented in RCC cell lines compared with that observed in HK2 cells (Figure 1G). Furthermore, we assessed the stability of circMYLK and observed that circMYLK was resistant to RNase R‐mediated degradation, unlike MYLK linear mRNA (Figure 1H).

**Table 1:** Patient information and clinicopathological characteristics of 71 patients with renal cell carcinoma. *P < .05 or **P < .01 was considered significant (chi‐square test between 2 groups)

Parameters	Group	Total	circMYLK expression		*P* value
			High	Low	
Gender	Male	49	37	12	.077
	Female	22	12	10	
Age (years)	<60	20	14	6	.787
	≥60	51	34	17	
Tumour size (cm)	<5 cm	44	36	8	.001
	≥5 cm	27	12	15	
Histological grade	Low	38	28	10	.24
	High	33	20	13	
T stage	1/2	46	30	16	.56
	3/4	25	18	7	
N stage	1	43	29	14	.971
	2	28	19	9	
Metastasis	Absent	40	31	9	.043
	Present	31	17	14	

### 3.2 Knockdown of cirCMYLK inhibits the growth and metastasis of RCC cells in vitro

**Figure 2:**
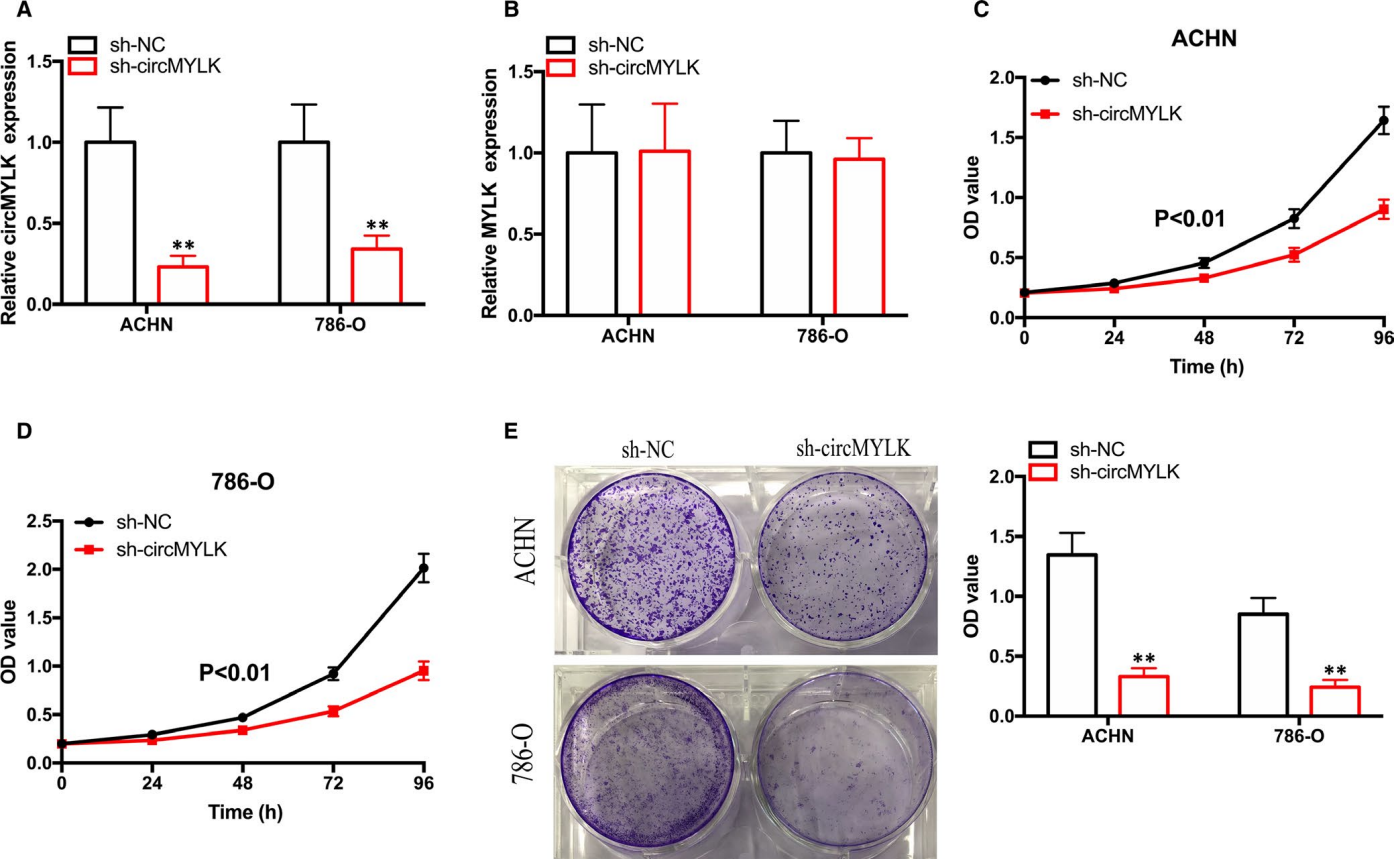
The silencing of circMYLK impairs cell proliferation. A and B, qRT‐PCR was performed to assess the expression of circMYLK and MYLK in RCC cells transfected with shRNA targeting circMYLK. C and D, CCK‐8 assays were performed to investigate the growth rate of RCC cells transfected with sh‐circMYLK. E, Colony formation assays were performed to assess the proliferation of RCC cells transfected with sh‐circMYLK. *P < .05 and **P < .01

To investigate the role of circMYLK in RCC cells, an shRNA targeting circMYLK (sh‐circMYLK) was used to suppress circMYLK expression in RCC cells. The qRT‐PCR results showed that circMYLK expression was effectively suppressed by sh‐circMYLK in RCC cells (Figure 2A). However, circMYLK silencing did not modulate MYLK mRNA expression (Figure 2B). The CCK‐8 assay results demonstrated that repression of circMYLK significantly impaired the proliferation ability of ACHN and 786‐O cells (Figure 2C and D). Furthermore, the colony formation assay results confirmed that the cloning capabilities of RCC cells were notably decreased by silencing circMYLK expression (Figure 2E).

**Figure 3:**
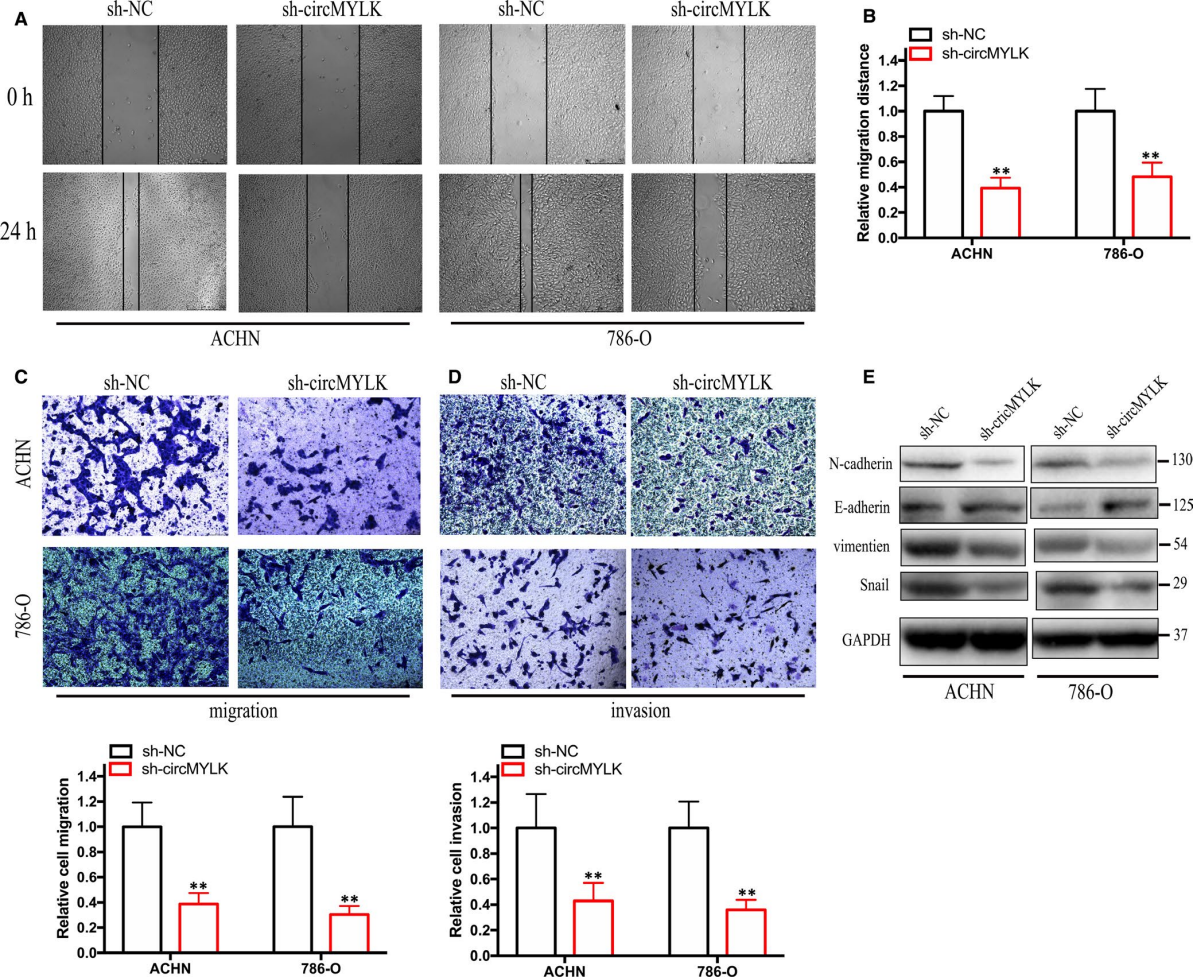
Knockdown of circMYLK represses the metastasis and EMT process of RCC cells. A‐C, The silencing of circMYLK inhibited the migration of ACHN and 786‐O cells. D, The silencing of circMYLK suppressed the invasion of ACHN and 786‐O cells. E, Western blotting was performed to assess the protein levels of EMT markers in ACHN and 786‐O cells after circMYLK depletion. *P < .05 and **P < .01

Subsequently, wound‐healing and Transwell assays were performed to measure the migration and invasion capabilities of RCC cells. Decreased RCC cell migration was observed after silencing circMYLK (Figure 3A‐C). The Transwell Matrigel invasion assay results demonstrated that silencing circMYLK impaired the invasion ability of RCC cells (Figure 3D). The epithelial‐mesenchymal transition (EMT) process is closely correlated with cell migration and invasion during the progression of cancers. As expected, suppression of circMYLK impaired the protein expression of mesenchymal markers (Snail, Vimentin and N‐cadherin) and up‐regulated that of the epithelial marker E‐cadherin (Figure 3E). Thus, these data revealed that circMYLK contributes to RCC growth and metastasis in vitro.

### 3.3 cirCMYLK abundantly sponges miR‐513a‐5p in rcc cells

**Figure 4:**
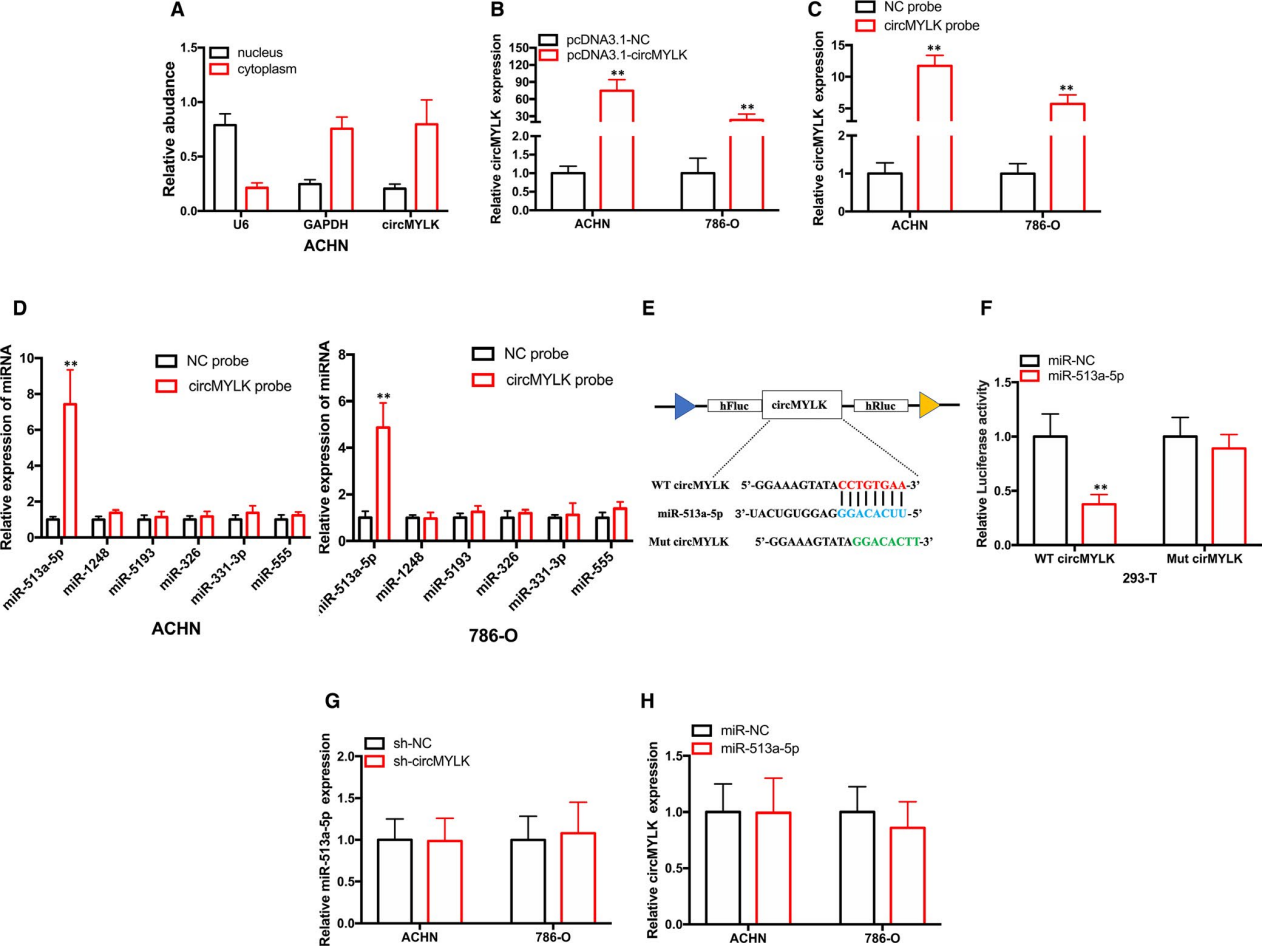
circMYLK can capture miR‐513a‐5p in RCC cells. A, circMYLK is predominantly distributed in the cytoplasm. B, The expression level of circMYLK in ACHN cells after transfection of pcDNA3.1‐circMYLK. C, qRT‐PCR results showing that circMYLK was enriched with a specific circMYLK probe in RCC cells. D, qRT‐PCR results showing that miR‐513a‐5p was pulled down by a specific circMYLK probe in RCC cells. E‐F, Relative luciferase activities in 293T cells were measured after transfection with luciferase reporter vectors and miR‐513a‐5p mimics. G, Analysis of miR‐513a‐5p expression in RCC cells transfected with sh‐circMYLK. H, Analysis of circMYLK expression in RCC cells transfected with miR‐513a‐5p mimics. *P < .05 and **P < .01

Next, cellular fractionation assays were performed to assess the subcellular localization of circMYLK. As shown in Figure 4A, circMYLK was enriched in the cytoplasmic fraction, as was observed in a previous study. circRNAs located in the cytoplasm may function as ‘miRNA sponges’ to capture miRNA. It has been reported that circMYLK acts as ‘miRNA sponge’ in the progression of various cancers. To determine whether circMYLK could sponge miRNAs in RCC cells, we predicted the potential binding of miRNAs with circMYLK using CircInteractome database. pcDNA3.1‐circMYLK plasmid was used to increase circMYLK expression in RCC cells. As shown in Figure 3B, the expression level of circMYLK was increased significantly after transfection of pcDNA3.1‐circMYLK plasmid. Furthermore, we used a circMYLK probe to perform RNA pull‐down assays to identify the miRNAs that bound to cirMYLK. As shown in Figure 4C, the specific circMYLK probe could pull‐down circMYLK in RCC cells upon the overexpression of circMYLK. In addition, only miR‐513a‐5p was pulled down by the circMYLK probe in RCC cells (Figure 4D). Furthermore, dual luciferase reporter assay results also demonstrated that circMYLK could directly bind to miR‐513a‐5p (Figure 4E and F). However, knockdown of circMYLK did not alter the expression of miR‐513‐5p (Figure 4G). In addition, the overexpression of miR‐513a‐5p did not modulate circMYLK expression (Figure 4H). These data suggest that circMYLK abundantly sponges miR‐513a‐5p in RCC cells.

### 3.4 miR‐513a‐5p notably impairs RCC proliferation and metastasis by suppressing VEGFC expression

**Figure 5:**
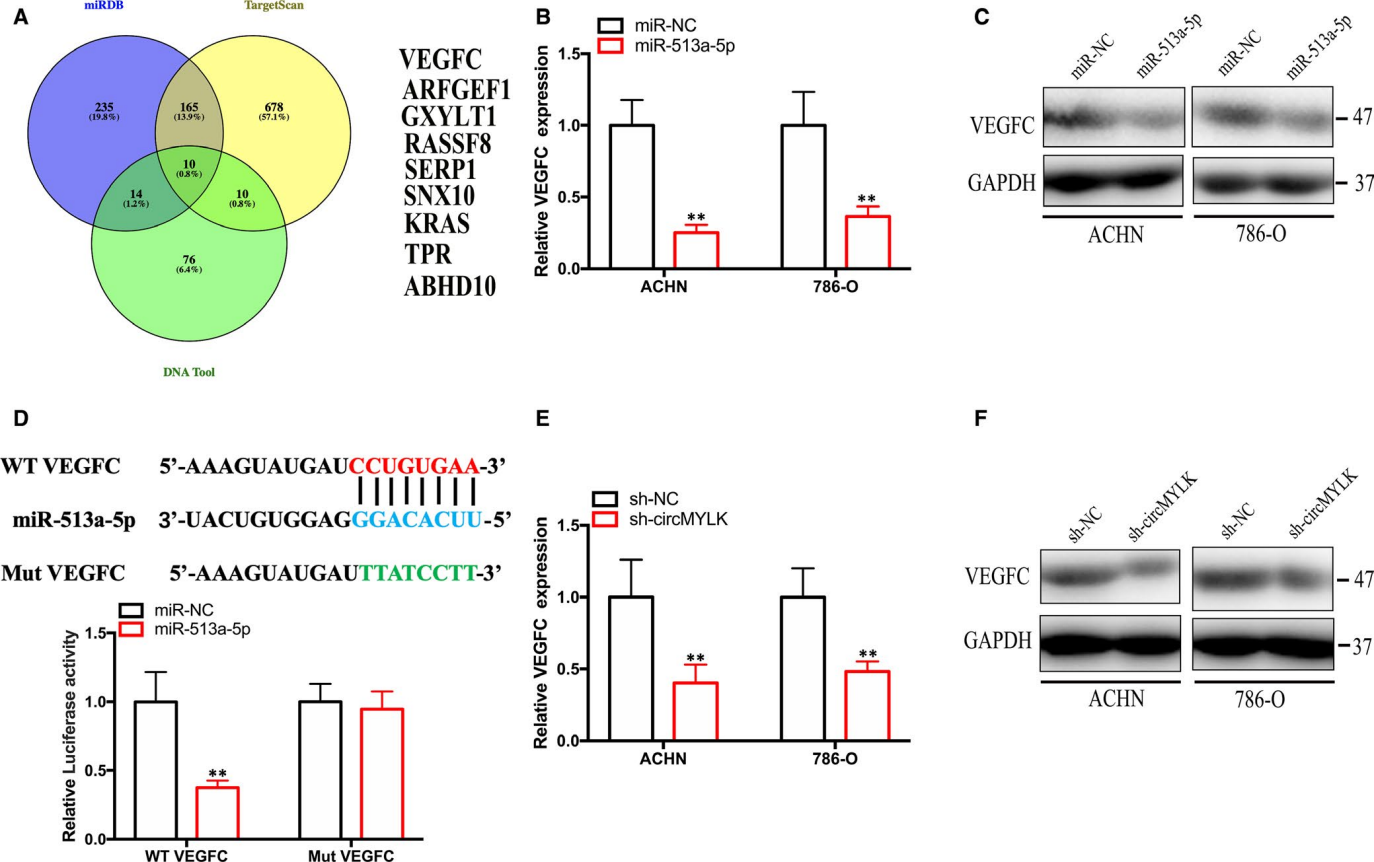
miR‐513a‐5p directly binds to VEGFC. A, Ten candidate genes associated with miR‐513a‐5p were identified using miRDB, TargetScan and the DNA Tool database. B‐C, Assessment of mRNA and protein expression of VEGFC in RCC cells transfected with miR‐513a‐5p mimics. D, The relative activities of luciferase in 293T cells were measured after transfection with luciferase reporter vectors containing VEGFC‐WT and miR‐513a‐5p mimics. E‐F, Detection of VEGFC mRNA and protein expression in RCC cells transfected with the sh‐circMYLK vector. *P < .05 and **P < .01

To identify the targets modulated by miR‐513a‐5p in RCC cells, we utilized the miRDB, TargetScan and DNA tool databases to predict the potential target genes. Ten candidate genes were obtained (Figure 5A). Interestingly, we discovered that miR‐513a‐5p overexpression decreased VEGFC mRNA and protein expression in RCC cells (Figure 5B and C). Furthermore, VEGFC and circMYLK share the same microRNA response element of miR‐513a‐5p, according to the TargetScan database. Subsequently, dual luciferase reporter assay results showed that the luciferase intensity of a luciferase reporter vector containing the WT 3′UTR sequence of VEGFC was notably decreased in cells transfected with miR‐513a‐5p mimics (Figure 5D). As expected, decreased circMYLK expression significantly suppressed VEGFC mRNA and protein expression (Figure 5E and F). These results suggest that miR‐513a‐5p can bind to the 3′UTR of VEGFC to inhibit VEGFC expression.

**Figure 6:**
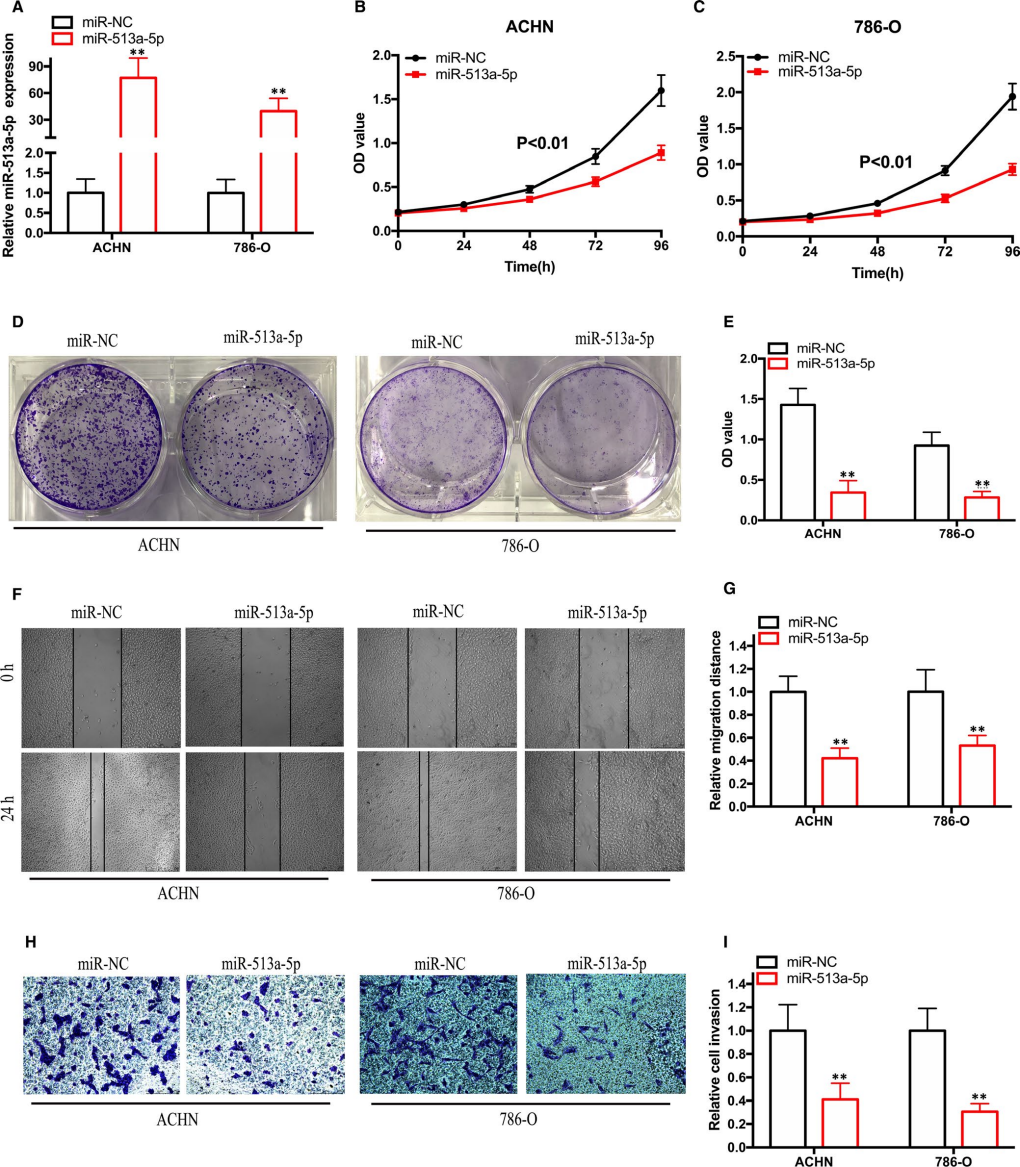
Overexpression of miR‐513a‐5p restrains RCC growth and metastasis. A, The level of miR‐513a‐5p expression in RCC cells transfected with miR‐513a‐5p mimics. B‐C, CCK‐8 assay results showing the growth rate of RCC cells transfected with miR‐513a‐5p mimics. D‐E, Colony formation assay results showing the proliferation ability of RCC cells transfected with miR‐513a‐5p mimics. F‐G, Wound‐healing assay results showing the migration ability of RCC cells transfected with miR‐513a‐5p mimics. H‐I, Transwell invasion assay results showing the invasion ability of RCC cells transfected with miR‐513a‐5p mimics. *P < .05 and **P < .01

Subsequently, the role of miR‐513a‐5p in RCC growth and metastasis was investigated. RCC cells were transfected with miR‐513a‐5p mimics or a negative control to assess the effect of miR‐513a‐5p. miR‐513a‐5p expression was significantly up‐regulated in ACHN and 786‐O cells transfected with miR‐513a‐5p mimics (Figure 6A). Furthermore, cell proliferation assay results demonstrated that augmented miR‐513a‐5p expression impaired the proliferation ability of RCC cells (Figure 6B‐E). Wound‐healing and Transwell invasion assay results demonstrated that miR‐513a‐5p overexpression inhibited RCC migration and invasion (Figure 6F‐I). These results indicate that miR‐513a‐5p can target VEGFC to suppress the development of RCC.

### 3.5 Overexpression of VEGFC reverses sh‐CRICMYLK‐mediated suppression of RCC proliferation and metastasis in vitro

**Figure 7:**
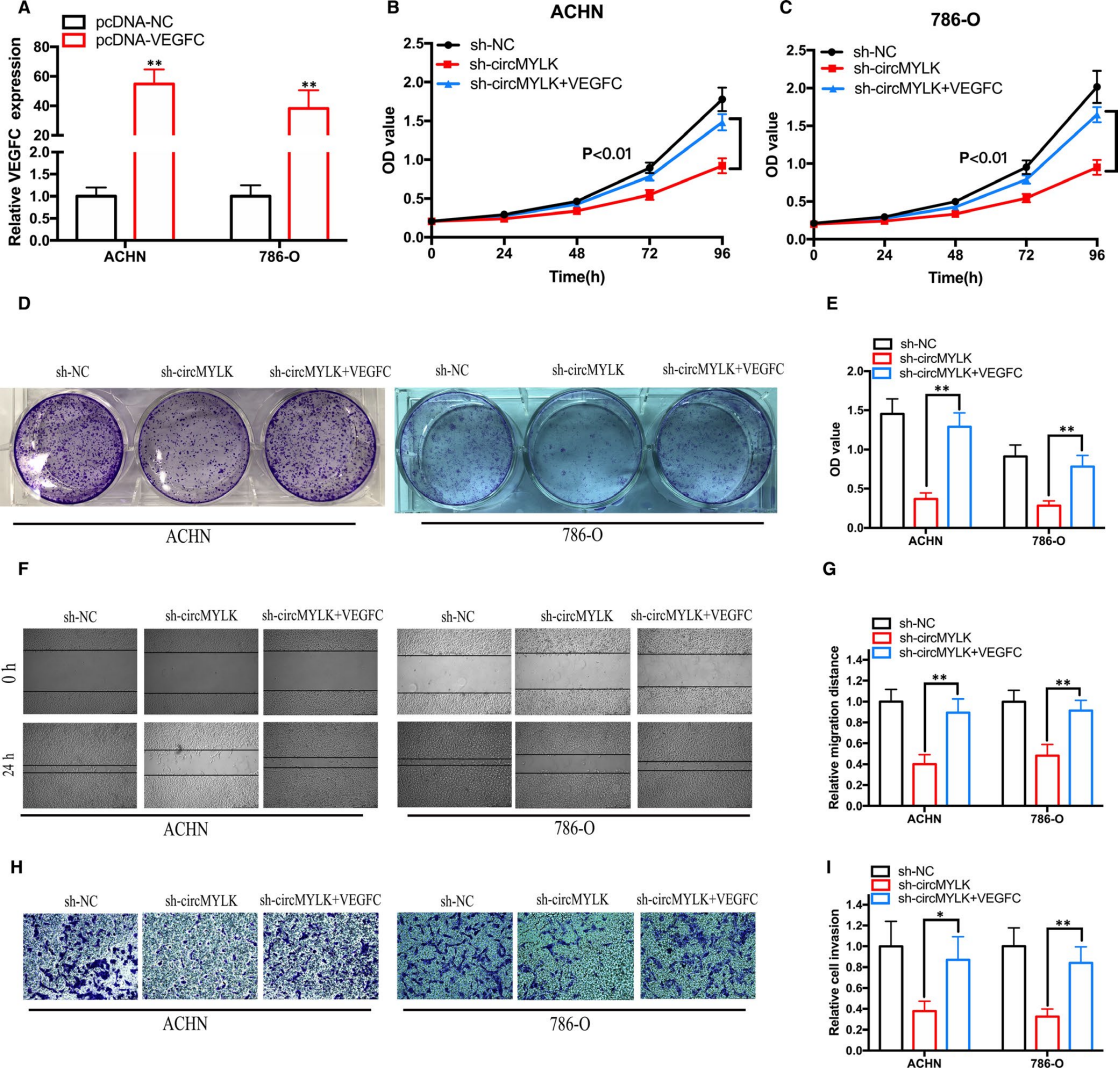
Overexpression of VEGFC reverses sh‐circMYLK‐induced inhibition of cell growth and metastasis in RCC cells. A, VEGFC expression was dramatically increased in RCC cells transfected with pcDNA3.1‐VEGFC. B‐E, Cell proliferation assay results demonstrated that circMYLK depletion restrained the proliferation capacity of RCC cells, and the inhibitory effect was reversed when cells were co‐transfected with pcDNA3.1‐VEGFC. F‐I, Would healing and Transwell Matrigel invasion assay results demonstrated that circMYLK depletion restrained the migration and invasion capacity of RCC cells, and the inhibitory effects were reversed when cells were co‐transfected with pcDNA3.1‐VEGFC. *P < .05 and **P < .01

To determine whether circMYLK promotes the proliferation and metastasis of RCC cells by modulating VEGFC expression, we co‐transfected sh‐circMYLK‐ and VEGFC‐expressing plasmids into RCC cells. The relative expression of VEGFC was dramatically augmented after transfection with the pcDNA3.1‐VEGFC plasmid in RCC cells (Figure 7A). Furthermore, the results of cell proliferation assays showed that VEGFC overexpression significantly reversed the inhibition of cell proliferation induced by silencing circMYLK (Figure 7B‐E). In addition, VEGFC overexpression significantly reversed the cell migration and invasion suppression induced by silencing circMYLK (Figure 7F‐I).

### 3.6 Knockdown of circMYLK inhibits the growth of RCC in vivo

**Figure 8:**
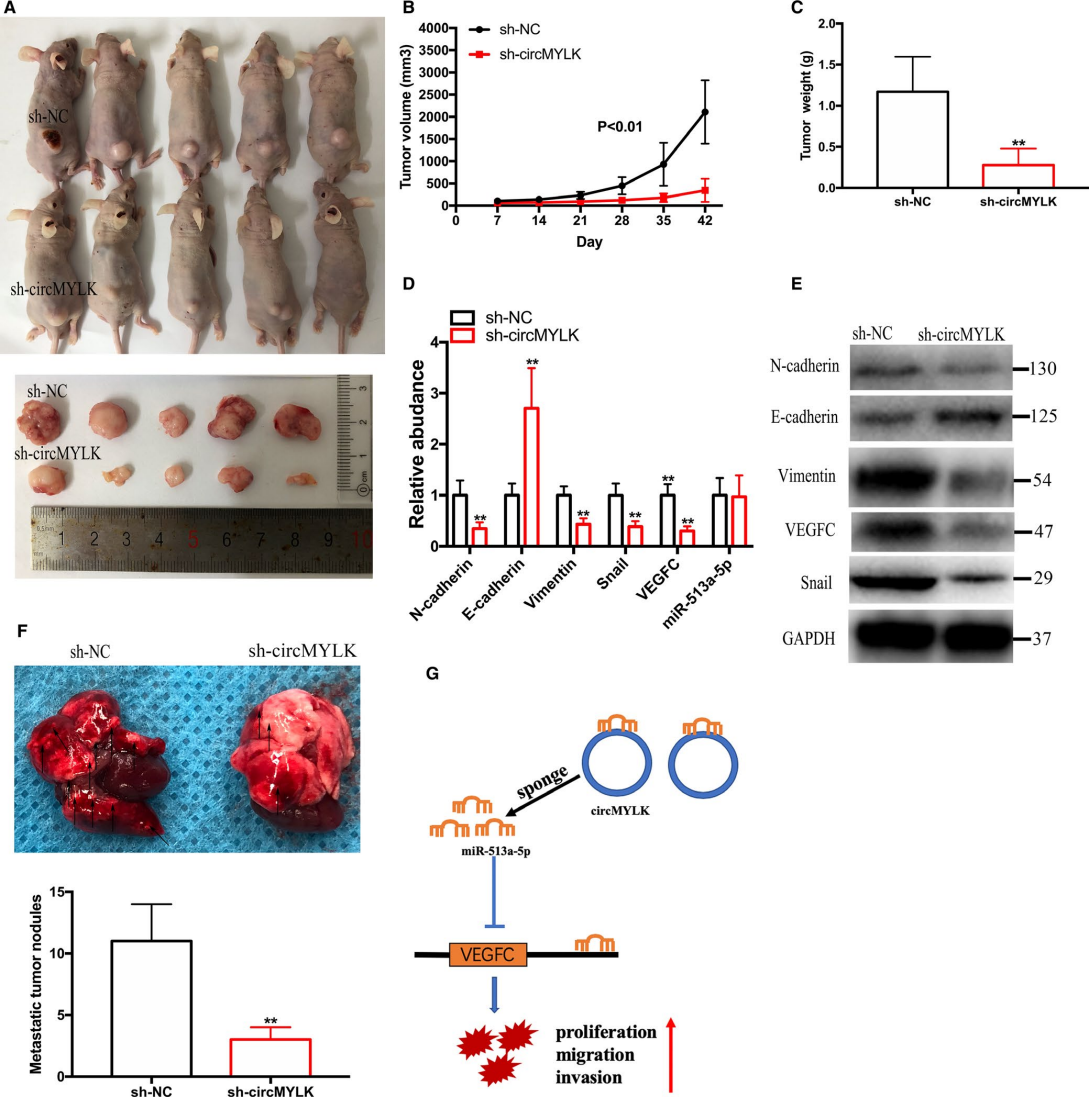
Depletion of circMYLK restrains RCC growth and metastasis in vivo. A, ACHN cells stably expressing sh‐circMYLK were injected into the backs of nude mice. Xenograft tumour models showed that tumours collected from circMYLK knockdown cells were smaller than those collected from the control cells. B, Tumour volumes were detected every week. C, Tumour weights were measured when the mice were sacrificed. D‐E, Depletion of circMYLK restrained the EMT process and VEGFC expression in vivo. F, Knockdown of circMYLK inhibited cell metastasis in vivo. G, Schematic illustration of the circMYLK/miR‐513a‐5p/VEGFC signalling. *P < .05 and **P < .01

To explore the effect of circMYLK in the growth of RCC in vivo, ACHN cells were stably transfected with sh‐circMYLK and then injected into the backs of nude mice to produce xenograft tumour models (Figure 8A). In the xenograft tumour model mice, we observed that circMYLK knockdown had a negative effect on the volumes and weights of tumours (Figure 8B and C). Furthermore, repression of circMYLK restrained the expression of mesenchymal markers (Snail, Vimentin and N‐cadherin), and VEGFC expression and increased the expression of the epithelial marker E‐cadherin in vivo (Figure 8D and E). However, knockdown of cricMYLK did not modulate miR‐513a‐5p expression in vivo. To investigate the effect of circMYLK in the metastasis of RCC cells in vivo, ACHN cells stably transfected with sh‐circMYLK were tail vein injected into mice. As shown in Figure 8F, silencing of circMYLK significantly inhibited RCC metastasis in vivo. Taken together, these results demonstrate that circMYLK contributes to RCC growth and metastasis by sponging miR‐513a‐5p to modulate VEGFC expression (Figure 8G).

## 4 DISCUSSION

With the extensive application of high‐throughput sequencing technology, numerous circRNAs have been identified in various tissues and cell lines.^15^, ^16^ Interestingly, a growing body of research has revealed that dysregulation of circRNAs causes a number of human diseases,^17^, ^18^ including autoimmune diseases,^19^, ^20^ cardiovascular diseases ^21^, ^22^ and especially human cancers.^4^, ^23^, ^24^ However, only a few circRNAs have been functionally identified with respect to RCC formation and development. In our study, the pathological and biological functions of cricMYLK in RCC were carefully investigated.

CircMYLK was observed to be derived from back‐splicing of exon 29 to exon 30 of MYLK mRNA, and the circMYLK gene is on chr3:123471177‐123512691. In addition, circMYLK is 376 nucleotides (nt) in length. CircMYLK has been shown to be an oncogene that promotes the progression of various cancers, including bladder cancer, prostate cancer, hepatocellular carcinoma and laryngeal squamous cell carcinoma. Zhong et al^13^ showed that circMYLK directly interacts with miR‐29a to modulate the VEGFA/VEGFR2 signalling pathway to promote the proliferation and metastasis of bladder cancer. Dai et al^14^ observed that circMYLK facilitates the progression of prostate cancer by suppressing miR‐29a expression. In addition, Li et al^25^ discovered that circMYLK can promote the growth and metastasis of hepatocellular carcinoma by sponging miR‐362‐3p and increasing Rab23 expression. In laryngeal squamous cell carcinoma, circMYLK can facilitate cell proliferation by modulating the microRNA‐195/cyclin D1 axis.^26^ However, the biological function and molecular mechanism of circMYLK in RCC have remained unknown.

This is the first study to investigate the biological function of circMYLK in RCC.

In our study, we showed that circMYLK was significantly up‐regulated in RCC tissues. Clinical correlation analysis indicated that circMYLK overexpression was positively correlated with larger tumour size, distance metastasis and poor prognosis of RCC patients. Further experiments demonstrated that silencing circMYLK restrained RCC growth and metastasis in vitro and in vivo. These data suggest that circMYLK is involved in RCC progression.

To investigate the molecular mechanism of circMYLK in the progression of RCC, we assessed the subcellular location of circMYLK and showed that circMYLK was predominately distributed in the cytoplasm. Mechanistically, circMYLK could sponge miR‐513‐5p to promote VEGFC expression. Knockdown of circMYLK did not alter the expression of miR‐513a‐5p, indicating that circMYLK acts as an ‘miRNA sponge’ of miR‐513a‐5p. As a competing endogenous RNA, the overexpression or knockdown circMYLK could not affect the total expression of miR‐513a‐5p, but only the unbound form of miR‐513a‐5p. In other words, circMYLK could not degrade miR‐513a‐5p at the post‐transcriptional level. CDR1a is a well‐known circRNA that binds to miR‐7 in neuronal tissues and contains 63 conserved binding sites for miR‐7. CDR1a directly absorbs miR‐7 to its microRNA response element and inhibits the activity of miR‐7. However, CDR1a cannot modulate the expression of miR‐7.^27^

The results of previous studies have indicated that miR‐513a‐5p is closely correlated with cellular sensitivity to cisplatin resistance and radioresistance in lung adenocarcinoma and osteosarcoma, respectively.^28^, ^29^ In HK2 cells, miR‐513a‐5p was shown to facilitate cell apoptosis induced by dichlorvos by suppressing Bcl‐2.^30^ In glioma, IGF‐1 could modulate miR‐513a‐5p expression to affect the NEDD4L/Wnt/β‐catenin signalling pathway and desensitize glioma cells to temozolomide.^31^, ^32^, ^33^ In our study, we showed that miR‐513a‐5p could impair RCC proliferation, migration and invasion, suggesting that miR‐513a‐5p can promote the proliferation and metastasis of RCC cells by suppressing VEGFC expression. The results of previous studies suggest that VEGFC acts as an oncogene to promote the proliferation, metastasis and angiogenesis in cancers.^34^, ^35^ In lung and colon cancer, suppression of VEGFC inhibits tumour growth, metastasis and EMT by expressing its receptor VEGFR3.^36^ In gastric cancer, VEGFC promotes cell metastasis and resistance to cisplatin by interacting with RhoGDI2.^37^ In RCC, up‐regulated VEGFC expression was correlated with increased distant metastasis and decreased overall survival.^38^ In our study, we observed that augmented VEGFC expression could apparently reverse the proliferation and metastasis inhibition induced by circMYLK suppression.

In summary, the results of our study suggest that circMYLK may be a promising prognosis marker and therapeutic target for the treatment of RCC. The novel regulatory network comprising the circMYLK/miR‐513a‐5p/VEGFC signalling pathway may provide a novel insight into the pathogenesis and development of RCC.

## CONFLICT OF INTEREST

All authors state that they have no competing interests.

## AUTHOR CONTRIBUTION

JFL designed the experiment, drafted the manuscript and performed data analysis. CCH and YFZ created the tables and figures and collected the RCC samples. JY and YTG provided fund for this study and supervised the project.

## ETHICAL APPROVAL

This study was approved by the ethics committee of Peking University Shenzhen Hospital, and written informed consents were obtained from each RCC patients.

## Supporting information

Table S1

## Data Availability

The data set(s) supporting the findings of this study are included within the article.
