# Corrigendum

**DOI:** 10.1111/jcmm.18079

**Published:** 2024-02-19

## Correction to Circular Rna Mylk Promotes Tumour Growth and Metastasis via Modulating Mir‐513a‐5p/Vegfc Signalling in Renal Cell Carcinoma

Li J, Huang CC, Zou Y, Jing Y, Gui Y. Circular RNA MYLK promotes tumour growth and metastasis via modulating miR‐513a‐5p/VEGFC signalling in renal cell carcinoma. *J Cell Mol Med*, 2020 Jun; 24(12):6609–6621. doi: 10.1111/jcmm.15308.

After publication of the article,^1^ a problem was identified in Figure 6. The authors observed that the results of wound‐healing assay in ACHN cells in Figure 6F were the same as that in AHCN cells in Figure 3A. They rechecked the original data and found that the results of wound‐healing assay in ACHN cells in Figure 3A were misplaced in Figure 6F.

These errors do not change the scientific conclusions of the article. The correct Figure 6F in ACHN cells is shown as below.

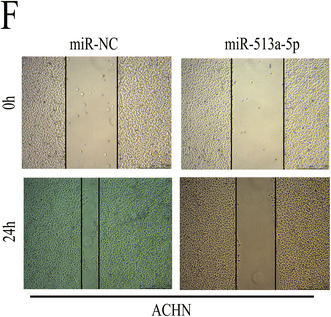

FIGURE 6 Overexpression of miR‐513a‐5p restrains RCC growth and metastasis. A, The level of miR‐513a‐5p expression in RCC cells transfected with miR‐513a‐5p mimics. B‐C, CCK‐8 assay results showing the growth rate of RCC cells transfected with miR‐513a‐5p mimics. D‐E, Colony formation assay results showing the proliferation ability of RCC cells transfected with miR‐513a‐5p mimics. F‐G, Wound‐healing assay results showing the migration ability of RCC cells transfected with miR‐513a‐5p mimics. H‐I, Transwell invasion assay results showing the invasion ability of RCC cells transfected with miR‐513a‐5p mimics. *p < 0.05 and **p < 0.01.
