# Supplementary material for: Circular RNA MYLK promotes tumour growth and metastasis via modulating miR‐513a‐5p/VEGFC signalling in renal cell carcinoma
Source: J Cell Mol Med. 2020 Apr 27;24(12):6609–21. doi: 10.1111/jcmm.15308 (PMC7299689; doi:10.1111/jcmm.15308)
Supplement: Supplementary file 1 — Table S1 [file JCMM-24-6609-s001.docx]

Supplementary Table 1. The primer sequences included in this study.

| Gene |  | Primer sequences (5’-3’) |
| --- | --- | --- |
| circMYLK | Forward | ﻿GCCTTGTGATTCATGCTGTCC |
|  | Reverse | ﻿CACATCCCCCATGGTCTTCT |
| MYLK | Forward | TGCCTCGTCACACATTTCCA |
|  | Reverse | GCAAAACTTCCCGCCCTTC |
| VEGFC | Forward | AACATCGCGGGGTGTTCTG |
|  | Reverse | CGGGTGTCAGGTAAAAGCCT |
| GAPDH | Forward | ﻿GGGAAACTGTGGCGTGAT |
|  | Reverse | ﻿AAGGGGTCATTGATGGCAAC |
| U6 | Forward | CTCGCTTCGGCAGCACA |
|  | Reverse | AACGCTTCACGAATTTGCGT |
| miR-513a-5p | Forward | CAGTTCACAGGGAGGTG |
| miR-1248 | Forward | AGACCTTCTTGTATAAGCACTGT |
| miR-5193 | Forward | GCAGTCCTCCTCTACCTCA |
| miR-326 | Forward | TCTGGGCCCTTCCTC |
| miR-331-3p | Forward | GCCCCTGGGCCT |
| miR-555 | Forward | CAGAGGGTAAGCTGAACCT |
